# *JSCAI* 2022: The Year in Review

**DOI:** 10.1016/j.jscai.2023.100605

**Published:** 2023-03-15

**Authors:** Alexandra J. Lansky

**Affiliations:** Section of Cardiovascular Medicine, Department of Internal Medicine, Yale School of Medicine, New Haven, Connecticut

**Keywords:** congenital heart disease, coronary disease, peripheral vascular disease, structural heart disease

This has been an exciting and truly enjoyable inaugural year. At the launch of the *JSCAI* in January 2022, we promised to deliver a new platform for our interventional cardiology community, to share and disseminate new clinical and scientific evidence that engages the interventional community at large and represents our Society for Cardiovascular Angiography & Interventions (SCAI) councils (coronary, peripheral, structural, and congenital), and to make this content accessible to all. By all measures, the first year of the *JSCAI* was a tremendous success, but I will let you judge for yourselves.

## Highlights of *JSCAI* 2022

In its inaugural year, *JSCAI* far exceeded all expectations for manuscripts submitted and published over 180 manuscripts; more than half were original research, meta-analyses, or comprehensive reviews. We published top interventional original science, with leading science evaluating intravascular lithotripsy, reporting the 1-year outcomes of the Disrupt CAD III study^1^ and the midterm outcomes of the randomized Disrupt PAD III trial,^2^ the outcomes of patients with STEMI and COVID-19 from the NACMI registry,^3^ and PCI in patients with heart failure,^4^ to name a few. The Pearls in Hemodynamics series led by Dr Morton J. Kern and Larry S. Dean is a feature essential to our readership. We have successfully delivered simultaneous publications with presentations at the SCAI 2022 Scientific Sessions and TCT 2022 and aim to do more to bring breakthrough science and innovation to life.

Our goal to reach a broad audience was extremely gratifying this first year: 20% of submitted manuscripts originated outside the United States, and 60% of our audience is international, representing 171 countries from Afghanistan to Zimbabwe with top interest coming from Europe, the United Kingdom, Canada, India, Brazil, Mexico, and Indonesia. We have just started to formally collaborate with other societies in the form of supplements to highlight the global reach of the *JSCAI*.

*JSCAI* articles have drawn broad interest, with hundreds of thousands of article requests and downloads through our platforms JSCAI.org and ScienceDirect. We are currently indexed in the Directory of Open Access Journals, and the PubMed Central indexing application is under review. We will be applying for indexing in the 2 largest search engines, Scopus and Clarivate, later this year. Our social media efforts continue to grow with more than 8200 Twitter followers of @MyJSCAI, with ongoing efforts to expand our reach through social media ambassadors.

## SCAI Standards and Guidelines

The SCAI has a vital commitment to establishing standards and guidelines led by the leading experts in interventional cardiology to establish best clinical practices, guide policy, and ultimately improve patient outcomes. The *JSCAI* is the home for all SCAI documents. By far, the SCAI SHOCK stage classification expert consensus update^5^ was the most requested document, followed by the SCAI guidelines for the management of patent foramen ovale,^6^ the SCAI position statement on best practices for percutaneous axillary arterial access and training,^7^ and the SCAI Expert Consensus statement on sex-specific considerations in myocardial revascularization.^8^ The SCAI publication committee continues to review and approve proposals for future publication and explores new partnerships with sister societies to assume a leading role in forming consensus within our expert interventional community.

## Partnership with *JACC: Cardiovascular Interventions*

Thanks in large part to the SCAI Immediate Past President Timothy D. Henry, the *JSCAI* was able to establish and implement a strong partnership with *JACC: Cardiovascular Interventions* only 6 months after the launch of the *JSCAI*. Many manuscripts have been referred from *JACC: Cardiovascular Interventions* to *JSCAI*, demonstrating that cross-society collaborations are not only possible but also powerful in strengthening our field. One outstanding manuscript published in the *JSCAI* because of this valuable partnership is authored by Weeraman et al,^9^ which was published with an accompanying editorial by Alkhunaizi and Burkhoff^10^ and a video conversation. Beyond offering authors the ability to seamlessly transfer a manuscript and shorten the timeline from submission to publication, our co-publication of standards and guidelines with the *JACC: Cardiovascular Interventions* extends the reach of SCAI content. We are grateful for this valuable collaboration.

## Conversations in Interventional Cardiology

In 2022, we recorded 31 videos as part of our Conversations in Interventional Cardiology series, covering all 4 SCAI member clinical interests. This unique method of extending the research beyond the pages allows us to continue the conversation with the *JSCAI* editors, authors, and other experts in the field. We plan to continue these videos as we explore what a journal can offer and the best ways to reach and serve the readership of the *JSCAI* and SCAI members.

## Editors and Reviewers

The outstanding editorial board and reviewers of the *JSCAI* kept their commitment to comprehensive, thoughtful, and competitive review and decision times that meet the expectations of our authors. The average time from manuscript submission to the first decision was 25 days last year; our goal is to shorten that decision time to 20 days. I want to personally thank our outstanding deputy, associate, and section editors, who have been steadfast in their commitment to deliver the highest standards of scientific content and without whom the *JSCAI* could not be successful. Our Statistical Editor, Helen Parise, has shouldered the bulk of the often overlooked but necessary statistical reviews that strengthen every manuscript we publish in the *JSCAI*. We have all enjoyed the dedication, flexibility, and collegiality of the SCAI team, led by Robert Bartel, Amanda Pettyjohn, and Chris Trimmer, and the Elsevier editorial and production teams, led by Jane Grochowski and Deanna Over, in supporting our efforts. Finally, Executive Editor Dominic Francese is the jewel of the *JSCAI*, tirelessly working to ensuring every manuscript is impeccable and delivered on time.

I am so grateful to all our reviewers who volunteer their precious free time, mostly for self-reward, in exchange for which they are recognized as valued members of our growing *JSCAI* editorial board. I would like to acknowledge our most active peer reviewers with our sincere thanks: John Blair, Sorin Brener, Adnan Chhatriwalla, Dmitriy Feldman, Beau Hawkins, John Hirshfeld, Morton J. Kern, Ki Park, Timir Paul, David Rizik, Michael Savage, Kimberly Skelding, Alex Truesdell, and Giora Weisz, among so many other contributors.

## Early Career Editorial Fellowship

Toward the end of 2022, we established the *JSCAI* Editorial Fellowship Program. This 1-year program will provide fellows-in-training and early career interventionalists the opportunity to enhance their skills as critical reviewers, obtain first-hand experience as editors, and have the opportunity to contribute editorials and conversations when appropriate. This program will allow our fellows to increase their exposure and involvement in the journal peer-review process, and I hope it will crystalize their academic interest as clinician investigators throughout their careers. Our first call resulted in 50 superbly qualified applicants from around the world, making our selection of the best from the best truly challenging. It gives me enormous pleasure to introduce our inaugural 2023 Fellowship participants who will be recognized at the SCAI 2023 Scientific Sessions: Mohammad Alnoor, Paul Bamford, Katherine Lee Chuy, Vikrant Jagadeesan, Aravdeep Jhand, Giorgio A. Medranda, Vinayak Nagaraja, Khajan Shah, and Saraschandra Vallabhajosyula. We are keen to mentor, open opportunities, and look forward to your contributions!

## Our 2023 Goals

Already, 2023 is off to a great start! My goals are to strengthen our visibility and international reach through joint educational sessions and society partnerships. Through this effort, I would like to increase our simultaneous publications with broader collaborations and develop themed issues with leading national and international faculty. I would also like to attract stronger contributions and representation from our congenital and peripheral councils and peers. It is my belief that through collaborations and partnerships, our field and academic footprint will grow stronger.
